# Constructing Highly Ordered Continuous BNNS Networks in COP Film to Achieve Excellent Thermal Conduction and Dielectric Performance

**DOI:** 10.3390/polym17233230

**Published:** 2025-12-04

**Authors:** Jialong Jiang, Yi Zheng, Yuan Ji, Hong Wu, Shaoyun Guo

**Affiliations:** The State Key Laboratory of Advanced Polymer Materials, Sichuan Provincial Engineering Research Center of Plastic/Rubber Complex Processing Technology, Polymer Research Institute of Sichuan University, Chengdu 610065, China

**Keywords:** boron nitride, continuous network, thermal conductivity, dielectric permittivity, dielectric loss

## Abstract

To meet the requirement of thermal management of modern electronic devices, polymer composites with high thermal conductivity (TC) and dielectric performance are nowadays in urgent demand. Herein, a highly ordered continuous network of boron nitride nano-sheet (BNNS) was constructed in cyclic olefin polymer (COP) films via the forced flow processing in the rubbery state (FFRS), melt-spinning, fiber-alignment, and hot-pressing procedures. The composites exhibited superior TC, low dielectric permittivity, and low dielectric loss simultaneously. The in-plane TC of the composites reached 3.92 W/(mK) when the content of BNNS was at 27 weight percentage (27 wt%), since the procedures improved the face-to-face contact between the BNNS (which was exfoliated, dispersed, and in-plane oriented during FFRS), enhancing the continuity of the BNNS thermally conductive network. Both the TC and the experimental results indicated the outstanding heat dissipation performance of the composites. Meanwhile, the dielectric permittivity and dielectric loss of the 27 wt% BNNS composites were 2.56 and 0.00085 at 10 GHz, respectively, lower than that of the COP-POE matrix. Moreover, the mechanical properties, water vapor permeability, and coefficient of thermal expansion of the composites were excellent. The composites with such highly ordered continuous networks are very promising in high-performance electronic devices.

## 1. Introduction

The continuous evolution of technology and the persistent pursuit of aesthetics led to severe challenges for thermal management systems. Overheating not only lowers device performance but shortens lifespans [1]. Thermal interface materials (TIMs), as the functional component of thermal management systems, are equipped between devices and heat sinks to eliminate interfacial air gaps and thus reduce thermal contact resistance [2,3], as well as to increase the interfacial thermal conductivity (TC) of the system. To meet the requirements of flexibility and high TC, TIMs typically consist of a flexible polymer matrix and highly thermally conductive fillers. However, achieving adequate TC in conventional TIMs requires excessive loadings of fillers [4,5,6,7,8,9], which are usually stiff, significantly damaging the flexibility and inducing extra interfacial air gaps, thus increasing the thermal resistance and possibly lowering the overall ability of thermal management [10]. Meanwhile, whereas the fifth generation (5G) wireless communication nowadays has been characterized by ultrahigh speed, low latency, massive capacity, and robust reliability, a severe problem of signal attenuation and heat generation was faced during 5G signal transmission, highlighting the urgent need for materials with low dielectric permittivity and dielectric loss [11,12] at ultrahigh frequency (10 GHz). Therefore, achieving high TC, low dielectric permittivity, and low dielectric loss of polymer composites at low filler loadings could be the keenest focus for developing high-performance TIMs.

For polymer matrices, cyclic olefin polymers (COP) refer to a kind of amorphous polymer with low dielectric permittivity, low dielectric loss, and low water vapor permeability [13], which suits the demand of high-performance TIMs well, and thus could be an ideal choice. As for fillers, hexagonal boron nitride (h-BN) is a two-dimensional ceramic filler with high TC (250–300 W/(mK)), low dielectric permittivity, and low dielectric loss simultaneously [14,15]. Furthermore, boron nitride nano-sheet (BNNS) exfoliated from h-BN has even a higher TC (2000 W/(mK)) than h-BN itself, and thus could be an ideal choice as well. However, reaggregation issues occur for BNNS, which is a nano-filler, and dispersion is necessary. Therefore, preparing and uniformly dispersing BNNS in the COP matrix is an effective method to achieve high TC, low dielectric permittivity, and low dielectric loss of polymer composites.

Currently, a variety of approaches have been developed for the preparation and dispersion of BNNS, including ball milling, liquid phase ultrasonic, and intercalation-assisted exfoliation [16]. However, whereas ball milling [17,18,19,20] and ultrasonic [21,22,23,24] approaches achieve preparation and dispersion of BNNS simultaneously, excessive reagent consumption and solvent abuse problems occur, leading to further treatment and intricate procedures. Worse still, the intercalation approach [25,26,27] not only faces such problems but requires further dispersion processing, leading to a more intricate procedure. Among these approaches, the forced flow processing in the rubbery state (FFRS) could be promising, which achieves preparation and dispersion of BNNS simultaneously in a simple, high-efficiency, and solvent-free way [28,29]. Furthermore, FFRS induces the in-plane orientation of BNNS, where the TC of BNNS is utilized totally within the plane of the composite film rather than in random directions, thus enhancing the in-plane TC of the film [30]. However, at low loadings, such dispersed BNNSs are usually hard to contact with each other, leading to discontinuous thermal pathways and a low in-plane TC of the film [30].

To construct continuous thermal pathways in the composites at low filler loadings, various approaches have been studied, such as external field induction, ice templating, and electrospinning [30]. However, electromagnetic field induction and ice templating are predominantly compatible with epoxy resins and silicone elastomers, while electrospinning requires strict control over polymer solubility and molecular weight. By contrast, melt-spinning followed by fiber alignment and hot-pressing represents an effective approach for composites with boron nitride nano-fillers. Zandieh [31] prepared PC/BNNT (boron nitride nano-tubes) composite fibers via melt-spinning, followed by in-plane alignment of the fibers and hot-pressing, thus constructing highly ordered continuous BNNT thermally conductive networks in the PC composites. Similarly, Du [32] employed fiber-alignment to enhance the continuity of BNNS thermally conductive networks.

In this work, we propose a highly efficient, solvent-free, and simple approach to construct highly ordered continuous BNNS networks in a COP-POE matrix via FFRS, melt-spinning, fiber-alignment, and hot-pressing procedures. POE was employed to enhance the fiber formability of the composites, where POE wt%:COP wt% was fixed at 3:70. The BNNS were first exfoliated from h-BN, dispersed, and in-plane oriented in the COP matrix, then overlapped with each other, constructing a highly ordered continuous BNNS thermally conductive network. The in-plane TC, dielectric permittivity, dielectric loss, mechanical properties, water vapor permeability, and coefficient of thermal expansion (CTE) of the composites showed large enhancements simultaneously over that of the COP-POE matrix when the loading of BNNS was 27 wt%, suggesting the capability of the composites as TIMs. This study provides new insights and methods for the solvent-free preparation of high-performance TIMs.

## 2. Materials and Methods

### 2.1. Materials

A cyclic olefin polymer (COP; Light transmittance (3 mmt) = 91%, T_g_ = 102 °C, elongation at break (23 °C) = 90%, water vapor permeability (40 °C, 90%RH (100 µm)) = 1.0 g/(m^2^·24 h); ZEONOR^®^-1020R; Zeon Corporation, Tokyo, Japan) was used as the matrix, together with h-BN (20–25 μm; Jiangxi Napu Co., Ltd., Shangrao, China) for fillers and polyolefin elastomer (POE; Tensile strength = 5.70 MPa, elongation at break = 1100%, T_g_ = −53.0 °C; Engage8660; Dow Chemical, Midland, MI, USA) to enhance fiber formability. Sample processing employed a rheometer (RM200C; Harbin Happ Electrical Technology Co., Ltd., Harbin, China) connected to an internal mixer (MIX-60C; Harbin Happ Electrical Technology Co., Ltd., Harbin, China), a two-roll mill (LRMR-S-150/EW; Labtech Engineering, Samut Prakan, Thailand), and a twin-screw extruder (SHJ20B; Nanjing Giant Machinery Co., Ltd., Nanjing, China). A micro melt-spinning system (MC5; Xplore, Sittard, The Netherlands) and a hot-pressing machine (HD-50T/400; Qingdao Huabo Machinery Technology Co., Ltd., Qingdao, China) were employed for the fiber and film preparation, respectively.

### 2.2. Preparation of the COP Composites

**Figure 1:**
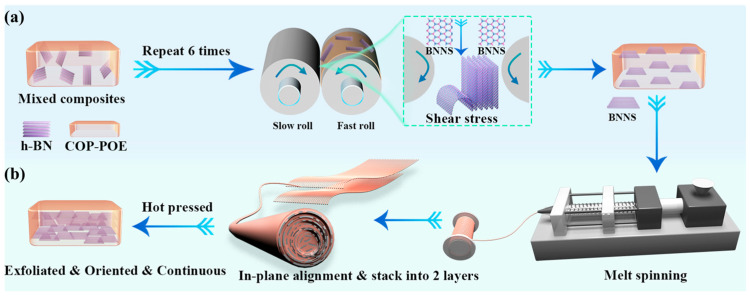
Schematic diagram of (**a**) the forced flow processing in the rubbery state (FFRS); (**b**) the melt-spinning, fiber-alignment, and hot-pressing.

For the exfoliated and oriented samples (the oriented samples), to eliminate adsorbed moisture, the COP, POE, and h-BN were pre-dried under vacuum at 60 °C for 12 h. Before melt compounding, the internal mixer was preheated to 240 °C in all three zones and equilibrated for at least 30 min to ensure temperature stability. The mixing chamber was then set to 30 rpm, and a total of 40 g of COP, POE, and h-BN was introduced. After 10 min of compounding, the COP-POE/h-BN blend was collected once the torque curve had reached a steady state. For subsequent two-roll milling, the front and rear rolls were adjusted to 130 °C and 125 °C, respectively, and equilibrated for at least 30 min. The roll gap was set to 0.15 mm, with roll speeds of 10 rpm (front) and 8 rpm (rear). A 50 g portion of the pre-compounded COP-POE/h-BN blend was fed between the rolls. Once flattened into a sheet, the material was removed, folded, and re-fed into the roll gap. This sequence was defined as one cycle of FFRS (Figure 1a) and was repeated six times. Finally, the samples were hot-pressed at 240 °C (both upper and lower plates) for 10 min. The samples were labeled with “X-O”, where X represents the wt% of boron nitride. For example, “24-O” refers to the exfoliated and oriented samples prepared from 24 wt% h-BN, with 72.88 wt% COP and 3.12 wt% of POE, where the POE wt%:COP wt% was fixed at 3:70.

For the exfoliated, oriented, and continuous samples (the continuous samples), after the six-time repeats of the sequence, the samples were not directly hot-pressed but cut into rectangular specimens (1 × 1 cm^2^). After thorough drying, the sample was processed in a twin-screw extruder to produce melt-spinning masterbatches. The temperature profile of the extruder was set to 200, 230, 240, and 240 °C for the feeding, compression, homogenization, and die zones, respectively, with a screw speed of 100 rpm. The resulting masterbatches were subsequently spun into COP-POE/BNNS composite fibers using a micro-scale melt-spinning apparatus. The temperature of the apparatus was maintained at 180, 240, and 240 °C for the feeding, melting, and die zones, respectively, with a screw speed of 10 rpm and a take-up velocity of 40 m/min. The fibers were aligned in-plane and hot-pressed at 240 °C for 10 min under a pressure of 10 MPa. The overall fabrication procedure is illustrated in Figure 1a,b. The samples were labeled with “X-C”, where X represents the wt% of boron nitride. For example, “27-C” refers to the exfoliated, oriented, and continuous samples prepared from 27 wt% h-BN, with 70 wt% COP and 3 wt% of POE, where the POE wt%:COP wt% was fixed at 3:70.

For directly mixed samples, h-BN, COP, and POE were compounded using a twin-screw extruder. The processing temperatures were set at 200 °C, 230 °C, 240 °C, and 240 °C for the feeding, compression, homogenization, and die zones, respectively, with a screw speed of 100 rpm. The resulting pellets were subsequently hot-pressed at 240 °C under a pressure of 10 MPa for 10 min. The samples were labeled with “X-M”, where X represents the wt% of boron nitride. For example, “24-M” refers to the directly mixed samples prepared from 24 wt% h-BN.

The formulations and detailed sample abbreviations are shown in Table S1.

### 2.3. Characterization

Fillers and cryo-fractured surfaces were sputter-coated and imaged via Scanning Electron Microscope (SEM, JSM5900-LV; Nippon Electronics Corporation, Tokyo, Japan) under 15 kV. One-dimensional X-ray Diffractometer (XRD, Ultima IV; Rigaku, Akishima, Japan) was conducted by Cu Kα radiation under 40 kV, 25 mA, and from 0° to 90° at 2°/min. TC were characterized via Laser flash analysis (LFA 467; Netzsch, Selb, Germany) at 25 °C, whereby the in-plane (sample diameter: 25.4 mm, thickness: 0.5 mm) thermal diffusivity (α) was measured. Specific heat capacity (C_p_) was characterized via differential scanning calorimetry (DSC, DSC3; METTLER, Zurich, Switzerland) from −10 to 100 °C, at 10 °C/min. Density (ρ) was characterized via a densitometer (GH-120M; Matsuhaku Scientific Instruments, Xiamen, China) via the drainage method at 25 °C. Thus, the respective TC (k) was calculated as $k=\alpha \times C_{p}\times \rho$. For thermal management performance, samples (20 × 20 × 0.5 ${mm}^{3}$) were sandwiched between a ceramic heater (15 W) and a heatsink, and surface temperature was monitored by an infrared camera (T620; FLIR, Hudson, NH, USA) during the heating and cooling process. For broadband dielectric measurements, the disks were sandwiched between two copper electrodes and examined using an impedance spectrometer (Concept50; Novocontrol GmbH, Montabaur, Germany) for dielectric permittivity and dielectric loss in the frequency range of 1 Hz to 1 MHz. In addition, square samples (5 cm × 5 cm) were prepared and characterized at microwave frequencies via the resonant cavity method using a vector network analyzer (model N5247A; Keysight Technologies, Bayan Lepas, Malaysia) for dielectric permittivity and dielectric loss at 10 GHz. Circular samples (diameter: 37 mm) were prepared and characterized via a water vapor permeability analyzer (C360H; Labthink, Jinan, China) under 35 °C and 90% relative humidity for water vapor permeabilities. CTE was characterized via a thermomechanical analyzer (TMA Q400EM; TA Instruments, New Castle, DE, USA), where rectangular samples (2 cm × 5 cm) were first annealed from 0 to 120 °C at a heating rate of 5 °C/min, followed by cooling at 20 °C/min under a nitrogen atmosphere. The applied preload and tensile force were carefully maintained between 1 and 5 mN. The tensile strength and elongation at break of the composites were characterized via a universal testing machine (Instron 5966; Instron, Norwood, MA, USA), where dumbbell-shaped samples (gauge length: 25 mm) were prepared and mounted in the testing grips and subsequently subjected to uniaxial tension at a crosshead speed of 50 mm/min in accordance with GB/T 1040-2006 [33].

## 3. Results and Discussion

### 3.1. Morphologies of the COP Composites

As discussed in the introduction, a variety of studies have suggested that aligning the composite fibers and subsequently processing them by hot-pressing is an effective approach to construct highly ordered filler networks within polymer composites. Following this concept, we aligned melt-spun COP-POE/BNNS composite fibers parallelly and in plane, followed by hot-pressing to construct highly in-plane oriented and continuous BNNS networks. The BNNS prepared via six circles of FFRS showed an average thickness of 55.7 nm and an average lateral size of 1.62 μm, as shown in Figure S1. An optical image of the alignment process is shown in Figure S2a (in supporting information), and the formation mechanism is illustrated in Figure S2b. Specifically, during fiber alignment, the diameter of each fiber is much smaller than the final film dimensions, while the BNNS content and distribution within each fiber remain nearly identical. Therefore, the parallel alignment step can be regarded as a process that ensures uniform BNNS dispersion within the sample. By contrast, samples prepared via merely FFRS suffer from uneven distribution of BNNS between the surface and interior of the sample due to the difference in shear velocities during FFRS. In addition, because BNNS have already been oriented along the fiber axis and formed a structure of quasi-coaxial circles with a common point of tangency during the melt-spinning, as shown in Figure S3b, the alignment process further promotes their in-plane orientation. Subsequently, during the hot-pressing of the aligned fibers, the BNNS within individual fibers are subjected to melt flow and compressive stresses. As a result, BNNS from both within and between adjacent fibers overlap with each other, forming a highly ordered BNNS network that is not only in-plane oriented but continuous in the COP composite.

**Figure 2:**
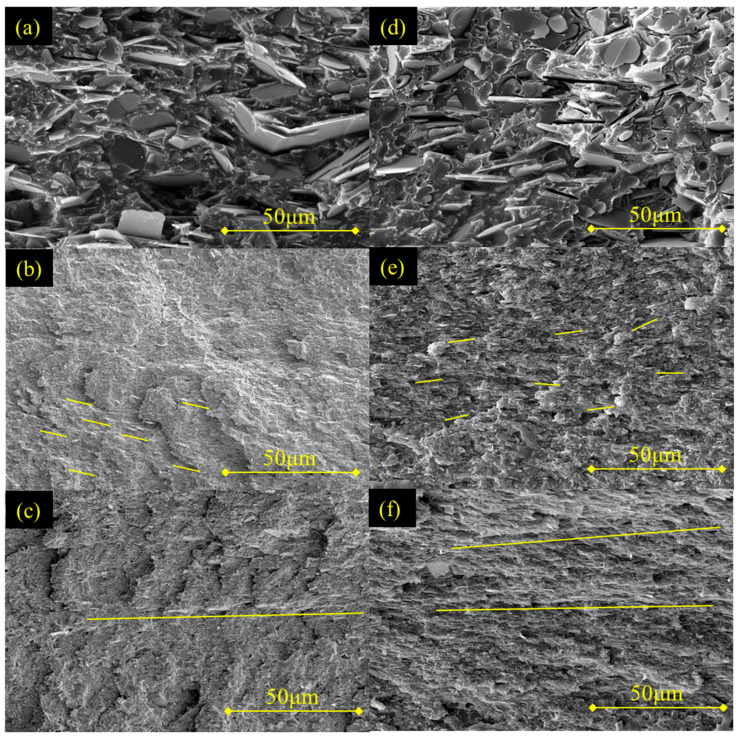
Cross-section SEM images of the COP composites: (**a**) 24-M; (**b**) 24-O; (**c**) 24-C; (**d**) 27-M; (**e**) 27-O; (**f**) 27-C.

Figure 2 shows the microstructure of the directly mixed, the oriented, and the continuous samples. The yellow lines highlighted the alignment of BN particles, where the orientation of the line corresponded to the orientation of the BN particles. In addition, short lines corresponded to separate BN particles (with either in-plane or random orientation), while long lines corresponded to in-plane oriented and continuous BN particles, forming continuous thermal pathways. As shown in Figure 2a–c, the 24-M sample displays large, randomly distributed h-BN particles with no discernible orientation. In the 24-O sample, the FFRS process exfoliates h-BN into BNNS and induces partial in-plane alignment; however, the gaps (highlighted in yellow) lead to weak interconnections between BNNS and discontinuous thermal pathways. By contrast, the 24-C sample exhibits a continuous and ordered BNNS thermally conductive network, wherein in-plane oriented BNNS overlap with each other, as highlighted in yellow, and form continuous thermal pathways. Figure 2d–f further compare the fracture cross-sections of the three composites at a higher filler loading (27 wt%). With increasing filler content, all samples show denser thermal pathways. Notably, the 27-C sample reveals a BNNS thermally conductive network that is not only dense but extremely highly ordered and continuous, with marvelously in-plane oriented BNNS effectively overlapping with each other in the COP composite. Such a hierarchical architecture of two-dimensional fillers is rarely achieved in polymer composites, providing pathways for phonon transport with superior efficiency. These SEM results unequivocally confirm that our integrated melt-spinning, fiber alignment, and hot-pressing approach constructs a highly ordered BNNS network within the COP matrix, which is the structural foundation for superior TC.

**Figure 3:**
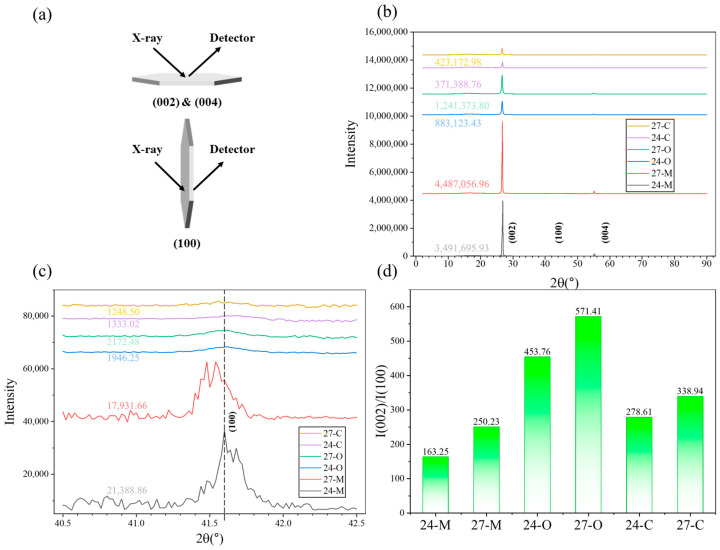
(**a**) Schematic diagram of the X-ray diffracted on BN particles corresponding to the horizontal (peak (002) and (004)) and vertical (peak (100)) orientation thereof; (**b**) XRD pattern of the COP composites from 0° to 90°, peaks and I(002) (Gaussian); (**c**) XRD pattern of the COP composites from 40.5° to 42.5°, (100) peak and I(100) (Gaussian); (**d**) I(002)/I(100) values of the COP composites.

To further investigate the microstructure of the COP composites, the XRD test was carried out on the directly mixed, the oriented, and the continuous samples (Figure 3).

As shown in Figure 3b, the intensity of peak (002) (I(002)) from the X-O and X-C samples was lower than that from the X-M samples at the same BN loading, indicating that the BN particles were successfully exfoliated into BNNS during the six cycles of the FFRS processing. Furthermore, the peak (002) from the X-O and X-C samples remained at an identical degree (around 26.7°) to that from the X-M samples, indicating that the FFRS processing introduced nearly no damage to the crystalline structure of BN, which remained unchanged during the exfoliation. The same applied to peak (100) (Figure 3c) as well, which remained at an identical degree (around 41.6°) across all samples. Moreover, the orientation degree of the BN in the COP composites could be evaluated via the ratio of the intensity of peak (002) against peak (100), i.e., I(002)/I(100), whereof the result is shown in Figure 3d. Both the 24-O and 27-O samples showed the highest value, corresponding to the highest in-plane orientation degree among the composites, indicating the validity of introducing the in-plane orientation to the BN particles via the six cycles of the FFRS process. In comparison, the 24-M and 27-M samples showed the lowest value, corresponding to the random orientation via the direct mixing. As for the 24-C and 27-C samples, the values were slightly lower than the 24-O and 27-O samples, corresponding to a lower in-plane orientation degree due to the disorientation effect during the melt spinning process. Nevertheless, the reorientation effect via the following fiber alignment and the hot-pressing process still ensured a higher in-plane orientation degree than that of the 24-M and 27-M samples, finally.

### 3.2. TC of the COP Composites

**Figure 4:**
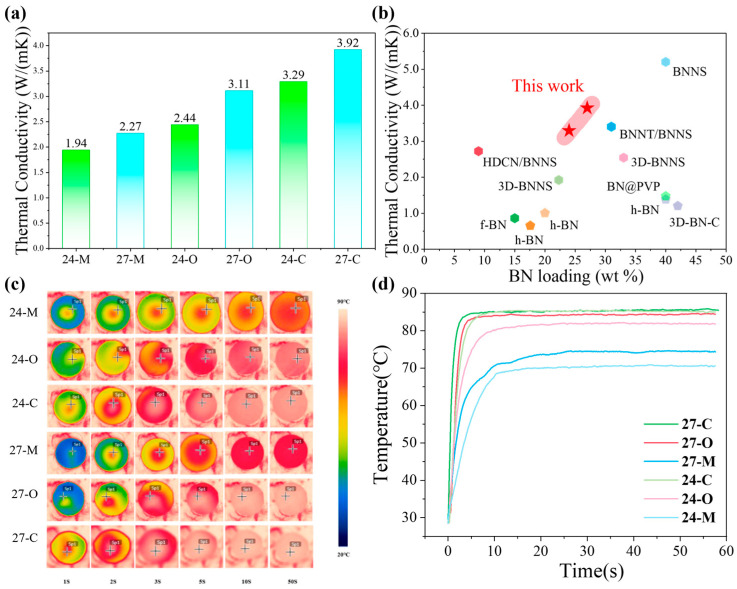
(**a**) In-plane TC of the COP composites; (**b**) in-plane TC of this work (marked as red stars) compared to other studies of BN composites [34,35,36,37,38,39,40,41,42,43]; (**c**) infrared thermal images at different times of the COP composites; (**d**) temperature–time curves of the COP composites.

The in-plane TC of the composites was characterized via DSC, the laser flash analysis, and a densitometry test, as shown in Figure 4a. A clear hierarchy of performance emerged. The 27-C sample showed the highest thermal conductivity, reaching 3.92 W/(mk), which corresponds to a 73% enhancement over the 27-M sample, highlighting the critical role of the highly ordered continuous BNNS thermally conductive network introduced by the melt-spinning and fiber alignment process.

Across both BN loadings investigated (24 and 27 wt%), a consistent trend was observed: directly blended and hot-pressed X-M specimens exhibited the lowest conductivities, whereas X-C samples achieved the highest. Notably, the superiority of this approach is particularly evident in the 24-C sample, which attained 3.29 W/(mk) and was higher than not only the 27-M sample (2.27 W/(mk)) but the 27-O sample (3.11 W/(mk)) despite containing less BNNS loading, corresponding to enhancements of 45% and 6%, respectively. The in-plane thermal conduction performance of the COP composite was highly determined by the in-plane orientation degree of BN fillers. For BN particles, the high TC remains along the lateral plane, while the TC in the longitudinal direction is far lower. In-plane orientation of BN particles corresponds to a parallel alignment of the lateral plane of BN particles to the lateral plane of the composite, guaranteeing a maximum utilization of the high TC of BN fillers in the in-plane direction, thus optimizing the in-plane thermal conduction performance of the COP composite. In addition, for exfoliated BNNS, the lateral size far exceeds the longitudinal thickness. In-plane orientation of BNNS corresponds to an identical in-plane alignment of the large-sized lateral plane of all BNNS, introducing a higher probability of overlapping one another BNNS to each BNNS, enhancing the continuity of the BNNS network and the thermal pathways in the in-plane direction, thus optimizing the in-plane thermal conduction performance of the COP composite. In addition, the COP-POE matrix contributed to the in-plane thermal conduction performance of the COP composite as well. For COP, which is a low-polarity and amorphous matrix, it provided a uniform environment that suppressed BNNS agglomeration and supported the in-plane orientation during the FFRS and melt-spinning process. As for POE, it contributed to the rheological properties of the matrix: the low incorporation thereof improved the melt strength and fiber formability of the COP-POE matrix, enabling the COP composite to be spun into fibers, thus enabling BNNS to be aligned and overlapped into a continuous thermally conductive network. Because COP and POE are mixed at a ratio of 70:3, the interfacial phonon scattering between COP and POE domains remains at the minimum level. Instead, it improves the construction of the BNNS network, which dominates the TC of the final material. Such TC results directly prove the superior TC of the highly ordered continuous BNNS network constructed in the COP composites.

To provide a comparison of the thermal management ability of the six composites as TIMs, in addition to DSC, laser flash analysis, and density measurements, we employed infrared thermography to monitor the surface temperature evolution of samples placed on a heating stage at 85 °C. As shown in Figure 4c,d, distinct differences are observed among the six composites in both the heating rate and the equilibrium surface temperature. Importantly, when correlated with TC, a clear correspondence emerges: composites with higher TC exhibit faster heating rates and higher steady-state surface temperatures. For example, sample 27-C reached 85 °C within just 5 s, highlighting its superior thermal management ability. By contrast, the 27-O sample with the same BN loading required up to 10 s to reach 85 °C, while the 27-M sample only reached 72 °C even after 50 s. Similar trends were consistently observed for the 24, 24-O, and 24-C samples. These results from infrared thermography are in excellent agreement with the measured in-plane TC, not only validating the reliability of the conductivity measurements but providing further confirmation of the highly ordered continuous BNNS thermally conductive network constructed in the COP composites.

### 3.3. Dielectric Performance of the COP Composites

**Figure 5:**
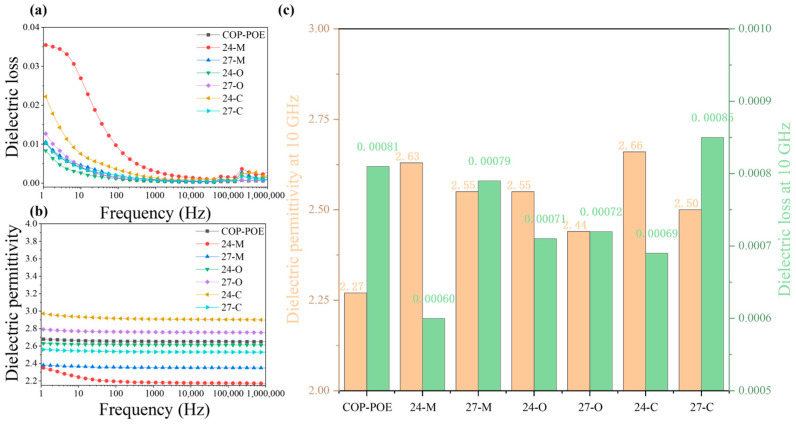
Dielectric performance of samples: (**a**) dielectric permittivity from 1 Hz to 1 MHz; (**b**) dielectric loss from 1 Hz to 1 MHz; (**c**) dielectric permittivity and dielectric loss at 10 GHz.

Dielectric properties of the X-C samples were characterized via a broadband impedance analyzer in the frequency range of 1 Hz–1 MHz, complemented by DSC, laser flash analysis, and densitometry. The results were benchmarked against the COP-POE matrix, X-O, and pristine X-M samples. As shown in Figure 5a, all seven materials exhibited a dielectric constant below 3 with negligible frequency dependence across the measured range. Particularly, the 24-C and 27-C samples showed dielectric permittivity of 2.98 and 2.56, respectively, positioning themselves among low dielectric permittivity materials. The dielectric loss spectra (Figure 5b) further revealed that all seven samples maintain losses below 0.001 in the 1 kHz–1 MHz region, with minimal variation among the composites. These results obtained from broadband impedance spectroscopy unambiguously demonstrate the outstanding dielectric performance of the COP composites.

### 3.4. Mechanical Properties of the COP Composites

**Figure 6:**
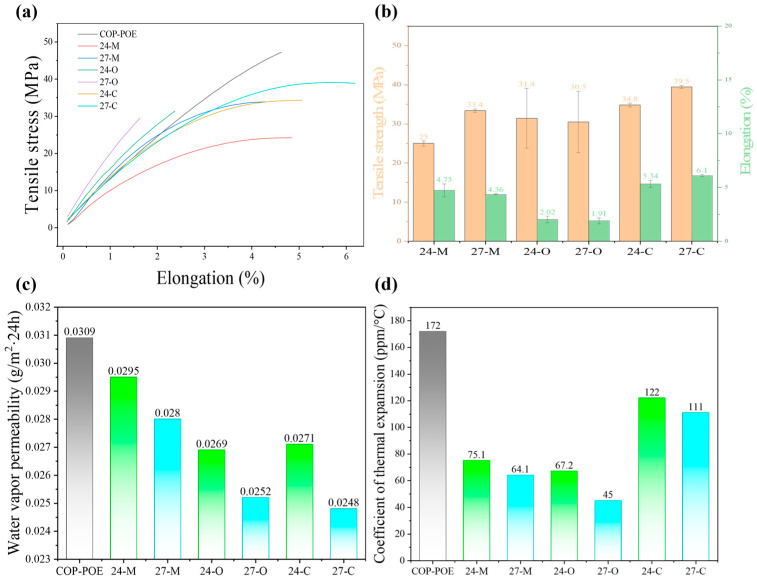
Further performance of samples: (**a**) tensile stress–elongation curve; (**b**) tensile strength and elongation at break; (**c**) water vapor permeability; (**d**) CTE.

The tensile strength and elongation at break of the COP composites are determined by not only the COP-POE matrix but the h-BN or BNNS fillers. As shown in Figure 6a,b, the melt-spun X-C samples showed large enhancements in both the tensile strength and elongation at break, compared to the X-M and X-O samples at identical BN loadings. For each composite, five replicate samples were tested, and the tensile strength was calculated as the average value of the results of the five replicates. Notably, the elongation at break of the 27-C sample showed a 219.37% enhancement based on the 27-O sample, indicating the contribution of the melt-spinning strategy to mitigating premature fractures, thus lowering the stress concentration during deformation. Beyond elongation at break, the tensile strength of 27-C reached 39.5 MPa, which is 30% higher than that of 27-O and 18% higher than that of 27, indicating the contribution of the highly ordered continuous BNNS network to reinforcing the COP-POE matrix.

To further investigate the mechanism of the melt-spinning and fiber-alignment strategies simultaneously enhancing the tensile strength and elongation at break, the POE phase in the X-M and X-C samples was removed by immersing the cryo-fractured samples in n-heptane (chromatographic grade; Aladdin Reagent Co., Ltd., Shanghai, China) at 60 °C for 12 h, subsequently characterized by SEM of the cross-sections. As shown in Figure S4, the POE phase in the X-C samples is not only more uniformly distributed but significantly smaller than that in the X-M samples. The size and dispersion of the POE phase are considered decisive in mechanical properties [44]: uniformly dispersed, fine POE particles effectively mitigate local stress concentrations and promote homogeneous plastic deformation of the COP matrix, thereby delaying premature failure. By contrast, large and irregularly distributed POE particles act as defect sites, which initiate crack propagation under load and even undergo interfacial debonding from the COP matrix, thereby compromising composite strength. Moreover, comparison of Figure S4a,c clearly showed that the POE phase in 27-C is smaller and more evenly dispersed than that in 24-C, providing the structural explanation of the higher tensile strength and elongation at break of 27-C than that of 24-C. Within the COP composites, POE formed finely dispersed elastomeric domains during melt-spinning, reducing stress concentrations and promoting uniform plastic deformation of the COP-POE matrix. Simultaneously, the continuous BNNS network (whereof construction was contributed by POE, as aforementioned) reinforced the matrix and distributed the load, both enhancing the mechanical performance of the COP composites.

These results not only indicate the simultaneous enhancement of the tensile strength and elongation at break of the COP composites due to the highly ordered continuous BNNS network but provide a compelling explanation of the mechanism thereof that the melt-spinning and fiber alignment strategies can optimize the POE phase morphology in the COP composites in both size and distribution.

### 3.5. Water Vapor Permeability and CTE of the COP Composites

The water vapor permeability of the X-C samples was characterized via a water vapor permeability analyzer and compared to the COP-POE, X-O, and X-M samples. As shown in Figure 6c, both h-BN and BNNS contributed to lowering water vapor permeabilities of the X-O and X-C samples compared to the COP-POE matrix, which could be owed to the “tortuous pathway” effect that effectively prolongs the diffusion path of water molecules. Among samples with identical BN loadings, the X-M samples exhibited the highest water vapor permeability, which could be due to the loose h-BN filler network that fails to establish an effective barrier, on the one hand. On the other hand, interfacial defects between COP and POE provided fast pathways for water vapor. At a BNNS loading of 24 wt%, the water vapor permeability of 24-C was slightly higher than that of 24-O; nevertheless, at the higher loading of 27 wt%, the water vapor permeability of 27-C decreased from 0.0252 to 0.0248 g/m^2^·24 h, lower than that of 27-O. This trend can be attributed both to the BNNS network in 27-C, which is denser than that in 24-C, and to the reduced POE fraction, which mitigates interfacial defects between COP and POE. These results indicate the ability of the water vapor barrier of the dense BNNS network, confirming the reliability of the COP composites in moisture environments.

The CTE of the X-C samples was characterized via a TMA and compared to the COP-POE, X-O, and X-M samples (Figure 6d). All six composites exhibit significantly lower CTE than the COP-POE matrix. Within each preparation method, composites with higher BN loadings showed reduced CTE, consistent with the intrinsically low thermal expansion of h-BN and BNNS, which restricted the mobility of COP chain segments. Notably, the X-C samples showed an increase in CTE compared to the corresponding X-M composites, which is because large POE particles tend to retain partial crystalline regions that suppress creep, whereas numerous finely dispersed POE domains may act cooperatively to amplify dimensional changes upon heating. Nevertheless, CTE of the X-C samples remained substantially lower than that of the COP-POE matrix. Particularly, 27-C achieves a CTE of 111 ppm/°C, which achieved a 55% reduction compared to that of the matrix (172 ppm/°C).

## 4. Conclusions

In summary, we successfully constructed highly ordered continuous BNNS networks in the COP composites via FFRS, melt-spinning, fiber alignment, and hot-pressing procedures. The SEM results indicated the BNNS thermally conductive network that is not only dense but extremely highly ordered and continuous, with marvelously in-plane oriented BNNS effectively overlapping with each other in the COP composite. Such a hierarchical architecture of two-dimensional fillers is rarely achieved in polymer composites, providing pathways for phonon transport with superior efficiency, leading to a TC of 3.92 W/(mK) when the BNNS loading was 27 wt% in the COP composites. As TIMs, the 27 wt% BNNS composites reached 85 °C within just 5 s, highlighting their superior thermal management ability. Last but not least, the dielectric permittivity and dielectric loss of the 27 wt% BNNS composites were 2.56 and 0.00085 at 10 GHz, respectively, lower than that of the COP-POE matrix. Moreover, the mechanical properties, water vapor permeability, and coefficient of thermal expansion of the 27 wt% BNNS composites were excellent, showing large enhancements based on the COP-POE matrix. Besides providing a new perspective for achieving excellent thermal conduction, this work suggests a new idea for the design and preparation of high-performance TIMs.

## Data Availability

The original contributions presented in this study are included in the article/Supplementary Material. Further inquiries can be directed to the corresponding authors.

## Supplementary Materials

The following supporting information can be downloaded at https://www.mdpi.com/article/10.3390/polym17233230/s1, Figure S1: The statistics of BNNS size after the forced flow processing in the rubbery state: (a) Thickness; (b) lateral size. Figure S2: (a) Optical image of the alignment process; (b) formation mechanism of highly in-plane oriented and continuous BNNS networks. Figure S3: SEM images of melt-spun 27-C fiber: (a) Surface; (b) cross-section. Figure S4: Cross-section SEM images of samples after POE phase removal: (a) 24-C; (b) 24-M; (c) 27-C; (d) 27-M. Table S1: Formulations of the COP composites.
