# Supplementary material for: Constructing Highly Ordered Continuous BNNS Networks in COP Film to Achieve Excellent Thermal Conduction and Dielectric Performance
Source: Polymers (Basel). 2025 Dec 4;17(23):3230. doi: 10.3390/polym17233230 (PMC12694120; doi:10.3390/polym17233230)
Supplement: Supplementary file 1 [file polymers-17-03230-s001.zip › polymers-3992734-supplementary.pdf]

Supporting Information

Constructing Highly Ordered Continuous BNNS Networks in COP Film to Achieve Excellent Thermal Conduction and Dielectric Performance

Jialong Jiang, Yi Zheng, Yuan Ji*, Yao Chen, Hong Wu*, Shaoyun Guo

The State Key Laboratory of Advanced Polymer Materials, Sichuan Provincial Engineering Research Center of Plastic/Rubber Complex Processing Technology, Polymer Research Institute of Sichuan University, Chengdu 610065, China

* Corresponding author, E-mail address: wh@scu.edu.cn, jy1996@scu.edu.cn

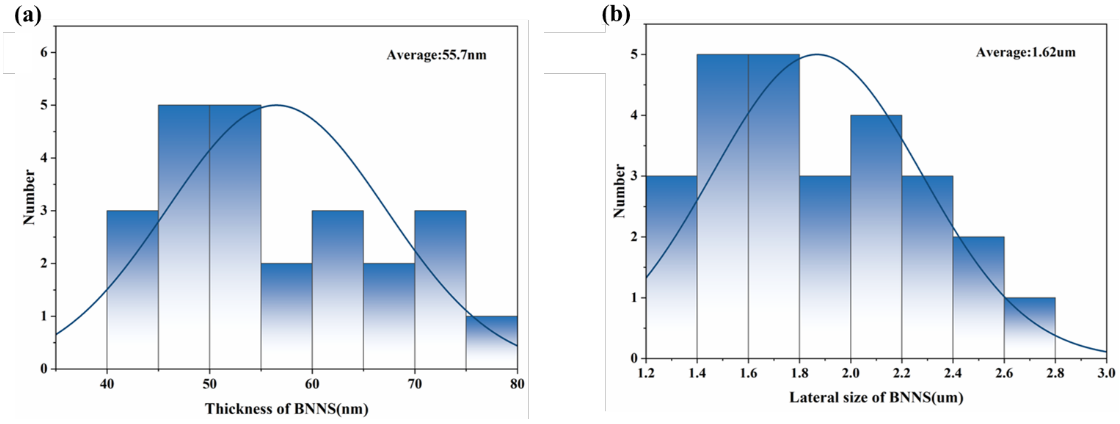

Figure S1: The statistics of BNNS size after the forced flow processing in the rubbery state: (a) Thickness; (b) Lateral size.

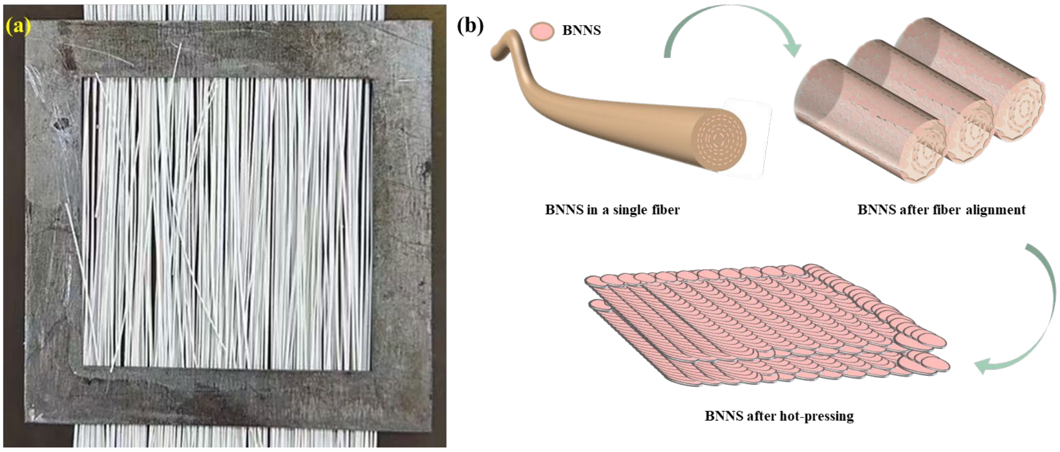

Figure S2: (a) Optical image of the alignment process; (b) Formation mechanism of highly in-plane oriented and continuous BNNS networks.

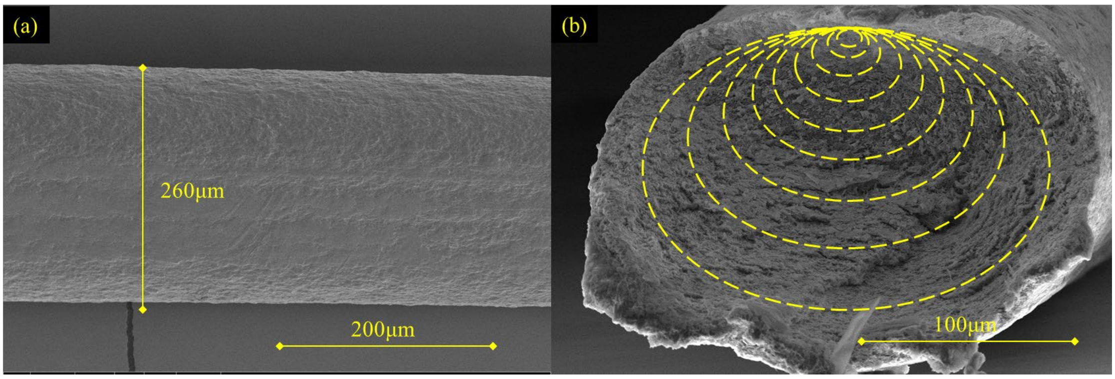

Figure S3: SEM images of melt-spun 27-C fiber: (a)Surface; (b) Cross-section, with the yellow curves indicating the alignment of quasi-coaxial circles with a common point of tangency of BNNS.

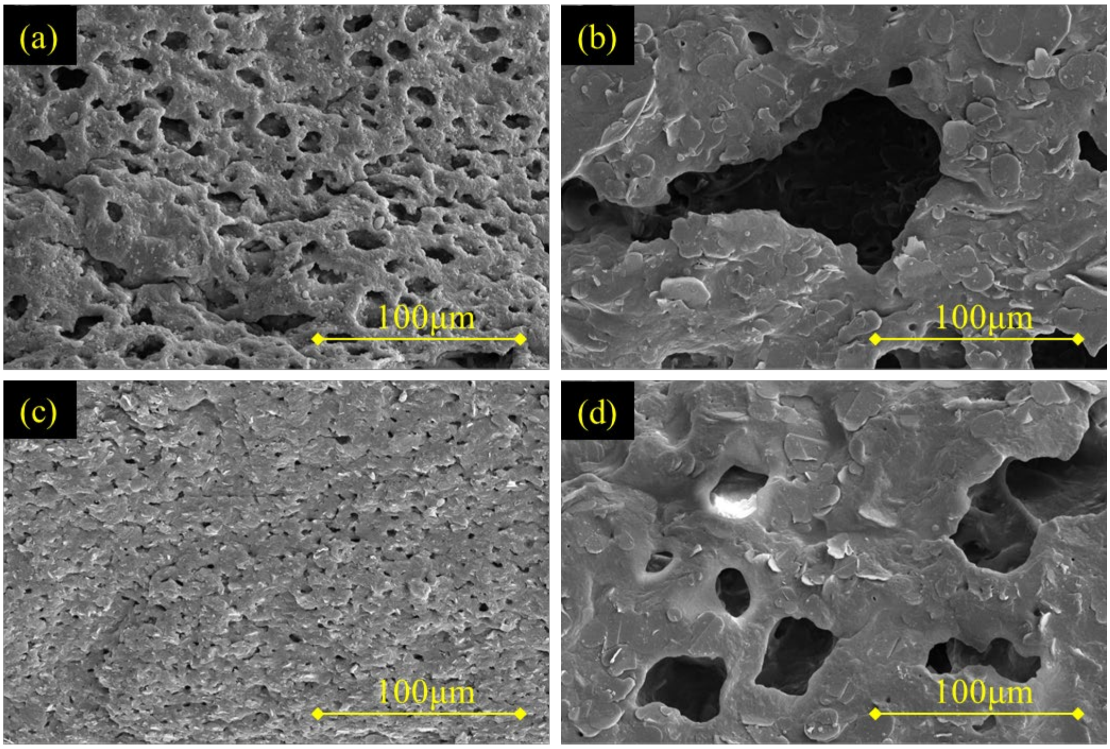

Figure S4: Cross-section SEM images of samples after POE phase removal: (a) 24-C; (b) 24-M; (c) 27-C; (d) 27-M.

Table S1: Formulations of the COP composites

Samples	BN wt%	COP wt%	POE wt%	Processing methods
24-M	24, h-BN	72.88	3.12	Directly mixing, hot pressing
27-M	27, h-BN	70	3	Directly mixing, hot pressing
24-O	24, BNNS	72.88	3.12	FFRS, hot pressing
27-O	27, BNNS	70	3	FFRS, hot pressing
24-C	24, BNNS	72.88	3.12	FFRS, Melt spinning, fiber alignment, hot pressing
27-C	27, BNNS	70	3	FFRS, Melt spinning, fiber alignment, hot pressing

(POE wt%:COP wt%=3:70)
